# A New Plan for the Treatment of Uterine Hemorrhages

**Published:** 1857-01

**Authors:** M. Dupierris

**Affiliations:** Havana, Cuba


ART. V.—*Observations on a New Plan for the Treatment of Uterine Hemorrhages.* By M. DUPIERRIS, Havana, Cuba.

I DO not intend to write a history of uterine hemorrhage, but of the treatment only; because this treatment is applicable to all kinds of uterine hemorrhage, no matter how caused. At the same time, it will not be amiss to offer a few general remarks on the subject.

The etymology of the word hemorrhage (*aima*, blood, and *reô*, I flow), expresses an action either pathological (such as occurs after the wounding of a vessel), or simply critical, as in certain constitutional hemorrhages, or, finally, presenting the characters of a physiological function, as happens in menstruation. Hemorrhages, however, may be divided into two general classes, normal and abnormal.

Many examples, one of which I have observed, are recorded, which prove that women, at a very advanced age, can be affected by menstruation for several months at a time, without any apparent injury to the economy. Hence, menstruation ought to be considered as a normal hemorrhage. Allow me to mention, in as few words as possible, the case of a lady of seventy-five years of age, who for three months consecutively had her courses as well-marked as in her youth, even to the exact number of days. This lady had fourteen children by her first husband. She was married a second time at the age of fifty-three; and, two years after, was delivered of a daughter, who was nineteen when I learned the above facts. Having been consulted by this lady, who could not believe that the discharge was natural, I persuaded her to do nothing; at the same time tranquillizing her by the recollection that her health not being affected, the flow could not be morbid.

But, when I assert that the menstrual flow is not like other hemorrhages, I must explain my meaning. Menstruation is not, properly speaking, a hemorrhage, that is to say, it is not a disease; and yet, I believe, that the periodical flow occurs in the same manner as a hemorrhage, because it is produced by the rupture of vessels. In other words, a certain process goes on in the ovaries, which lasts fifteen days in some women, and a month in nearly all. This process occasions an afflux of blood towards this part; the Graafian vesicles are developed and open; the egg is expelled by the mass of blood which flows to the part, and the discharge continues to push the egg, which arrives at its destination when menstruation ceases. The function, which is manifested by the periodical flow of blood, is the one which prepares the elements for the reproduction of the human kind. Is this the mode of formation of menstruation? I will try to prove it, by adducing some facts in support of my opinion. Some authors believe that the period at which woman is most apt to conceive, is immediately after the termination of her courses. I agree with them. Comparative physiology throws light on the subject. Twice a year the bitch is affected by a bloody discharge from the sexual parts, which lasts for three or four days. This discharge is preceded by a tumefaction of these parts, which is a sign that fecundation can occur. Since this is the only time in which sexual intercourse can occur, ought it not to be concluded that the eggs leave the ovary, and that the blood which flows, as well as that which causes the tumefaction of the external parts, was brought there in a manner very similar to that already mentioned. But since this function continues for a longer time—for this process requires six months for its completion—it is easy to understand that the dilatation of the cellular tissue, going on slowly, will allow a greater tumefaction; while in woman, the function which renders her capable of conceiving is completed every month; so that the afflux of blood has not the time to produce the same degree of congestion as in the former. For the same reason the genital organs are not tumefied at the menstrual period. It appears to me, also worthy of remark, that if the flow of blood from woman did not find an issue, furnished by the rupture of the Graafian vesicle, it would be more than sufficient to congest all the tissues of the organs of generation, and then the external flow would be less extensive, since the portion thus effused, would be more likely to be reabsorbed than poured into the uterine cavity.

If what I have just said is true, we may safely infer that menstrual blood does not differ from blood obtained from a ruptured vessel, except in the addition of epithelial pavement-cells and of the mucous fluids, proceeding from the genital organs, which combine with the serum of the blood. This takes place, as well in the bloody discharge occurring in organic diseases of the uterus, and in the commencement of hemorrhage, as in menstruation. Now the analysis of the blood could not afford means of distinguishing between a menstrual discharge and a hemorrhage, especially where menstruation lasts several days, or where a hemorrhage is slight.

It has also been said, that menstrual blood has not the power of coagulation. Clots are not formed when the courses are slight; but it is not thus in cases where they are abundant; and I have seen a number of instances in which clots of moderate size were formed, without any symptoms of hemorrhage being discoverable. This sign, so often pointed out as an essential diagnosis, is, in my opinion, of little value. In examining the symptoms, it ought not to be forgotten, that the menstrual flow can be a great deal more abundant than usual, without being caused by hemorrhage; because this greater abundance may be the result of the non-appearance or the diminution of the courses of the preceding month. I must also say, that there are uterine hemorrhages which take on the periodic character of menstruation, and are confounded with them, no matter how they may be produced.

I shall now proceed to establish the diagnosis of uterine hemorrhages.

But before I enter into the description of the symptoms of this bloody discharge, I will mention that I do not make any difference in regard to the mode of production of hemorrhages. These are all the consequence of the rupture of bloodvessels, whatever may be the exciting cause. Thus I do not admit hemorrhage by exhalation, nor its subdivisions, into active or passive hemorrhages. Physiology and anatomy sustain the opinion which I here advance; hence, in certain cases the rupture of the vessels is produced by the progressive ulceration of the surface of the tissue affected, as in cancerous or other tumors; in other instances it is the afflux of blood into the capillaries which produces their rupture, &c. &c.

It has been said that hemorrhages which are not puerperal are hardly ever observed before puberty, and still more rarely in young virgin women. The same author refers besides to the opinion that in young girls, after puberty, the discharge is generally symptomatic of an abortion.* I do not wish, however, to influence my readers in such a way as to make them forget that such may occasionally be the case; but, an opinion so exclusive exposes the practitioner to form his judgment too hastily in a question which touches on legal medicine, and to become an instrument of sorrow instead of consolation to humanity. I have, on several occasions, observed uterine hemorrhages in young women still virgins. I watched them with that interest which so delicate a subject inspires; and I positively affirm that the disease in girls at puberty can take place with a certain frequency, without its being established as a rule that it is produced by abortion. On the contrary, I consider these cases as the exceptions. The greatest number of uterine hemorrhages which take place at different periods of life are not observed in young girls; it is in the uterine period of life that they occur most frequently; especially when those large discharges which endanger life take place. But those oftenest attacked by idiopathic hemorrhages of a slow and continued form are women who have reached the critical age.

*Causes of Uterine Hemorrhages.*—Uterine hemorrhages can be caused not only by everything that may produce hemorrhages generally, but also by circumstances which act specially on the uterus. They occur principally in women of nervous or sanguineous temperament, particularly if they have had several miscarriages, and if they have used excitants and emmenagogues to excess. Moral emotions, the excitation of the genitalia, the presence of a foreign body, as a polypus, a fibrous or osseous substance, a calculus, a cancerous affection, &c., and, during pregnancy, the adhesion of the placenta after labor, the partial or total separation of the placenta accompanied by inertia of the uterus, and the tearing off of a part of the placenta—an example of

* I possess too many facts which prove the contrary to this, to allow me to think this opinion otherwise than very hazardous.

which I will describe a little farther on—should also be ranked among the causes of these discharges.

*Symptoms.*—The quantity of blood lost during menstruation not being the same in all women, it is important to know the symptoms by which the normal flow may be distinguished from the abnormal. The quantity of blood discharged varies according to the disposition of individuals, their manner of living, and the influences which are susceptible of acting upon the whole system, but particularly on the uterus. I must once more call attention to the variations which may occur as regards the quantity of blood at different times, without its being injurious to health. The loss can take place very slowly or very rapidly, or through the too frequent return of the courses: this always constitutes a hemorrhagic flow.

I have observed this kind of hemorrhage most frequently at the critical age; in women or girls of lymphatic temperament; those of great moral sensibility; those given up to debauchery; or in those who have experienced abortions.

The symptoms which precede hemorrhage are generally a feeling of lassitude, vertigo, pain in the head, in the lumbar region, and in the abdomen. Those which accompany the complaint are great debility, paleness of the face, gaping, sighs, dyspnœa, fainting, coldness of the extremities, convulsions, cold sweats, diminution or absence of pulse, &c.

*Pregnancy.*—There are women who continue to menstruate during the whole, or at least during several months, of pregnancy. This flow might be taken for an abnormal hemorrhage; but this mistake can be avoided, if the time at which the courses are generally seen, the symptoms which ordinarily precede them, the manner in which they take place, and finally the degree of influence acting on the economy, are taken into consideration. Hemorrhages depending upon the rupture of small vessels, or on the separation of a small portion of the placenta, may pass unperceived, on account of the small quantity of blood which may remain effused between the uterus and the coverings of the fœtus. This circumstance is very rare, and it is hardly necessary to mention it.

The symptoms which accompany hemorrhages during pregnancy, are those indicating expulsive contractions, namely, alternate calms and pains in the lumbar and hypogastric regions. The blood, which, at first, during the pains, flowed but slowly, increases very much when they become moderated, the hemorrhage thus continuing its course. The loss can cause death if the labor or the delivery of the placenta is retarded. The nearer the time at which the hemorrhage occurs approaches the moment of delivery, the more abundant, and, in consequence, the more dangerous, it will be. The general symptoms of hemorrhage, and those of uterine hemorrhage in particular, will serve to distinguish the nature of this disease.

*Hemorrhage of Women in Labor.*—Woman can go through the whole period of pregnancy without meeting with any accident, and yet be attacked with a uterine hemorrhage in the midst of labor. Two causes alone can produce the accident: first, a wound of the uterus, or a greater or smaller separation of the placenta, whether the latter be situated on the sides, on the fundus, or at the neck of the uterus. The symptoms which indicate this hemorrhage are particularly a great feebleness of the pulse, without any other manifest reason, sighing, and coldness of the extremities. When, during the absence of the pain, the accoucheur pushes back the head of the fœtus, or when labor ends, the blood rushes out in great quantities through the vagina; but these hemorrhages, dangerous as they are, may be arrested. Not so with those which are caused by the rupture of the uterus,—they are generally fatal. It is only by the symptoms that we are enabled to distinguish these latter cases, and they are not generally manifested until the blood has escaped into the peritoneum. Authors thus describe these unfortunate cases: in the midst of a violent pain the woman screams, and experiences the feeling of an internal rupture; at the same moment, or a short time after, she feels an inexpressible anxiety, faints, suffers from convulsions, and expires without losing much blood from the vagina.

*Hemorrhage after Labor.*—When, in women weakened by too early a labor, the uterus is distended beyond measure by the product of conception, or when, in consequence of the prolongation of labor, the remaining strength of the patient is destroyed, and when the afterbirth is expelled very soon after the child, an inertia of the womb will often result. Under these circumstances, the sinuses being open, hemorrhage naturally follows. It will be admitted that the part least susceptible of contraction is that at which the placenta was attached. In fact, this part, the surface of which is uneven, is also strongly engorged; and this particularity may be the cause of a mistake on the part of the physician. Thus the uterus, in contracting more actively in every other part than in the tumefied, the latter will form a hernia in the uterine cavity, and the torn utero-placental vessels comprised in this mass being compressed for the moment, will furnish a very small quantity of blood; but if the contraction is produced by a spasm, the latter having ceased, a hemorrhage, either apparent or latent, will be produced. If the spasm has been of short duration, the utero-placental vessels, compressed for a very short period, will not be able to contract. Hence, the tendency of the sanguineous afflux towards this region persisting, the hemorrhage will be violent, the more so as it is probable that the inertia of the uterus will follow the cessation of the spasm. Should the effects of the spasm be partial; in other words, should the latter affect only the neck of the uterus, the blood will be retained, and a clot will be formed near that part. This constitutes the latent hemorrhage. It may be conceived that a large quantity of blood may be accumulated in the cavity of the uterus, before sufficient signs can lead to the discovery of this affection. The symptoms which will be first manifested are pain, similar to that occurring when the uterus is in the act of expelling a foreign body; but even this symptom may only take place after the quantity has put the life of the patient in danger. Attention must therefore be paid to the general symptoms of hemorrhage.

The placenta may be adherent, but lacerated somewhere,* and an immense hemorrhage may occur, especially as the detachment of the placenta will take place more slowly, because the inertia of the uterus accompanies this condition. This hemorrhage will continue until the artificial separation of the placenta has taken place, and until the inertia has ceased.

Uterine hemorrhage may also be the effect of the want of power in the uterus to contract, owing to the large size of the placenta, the presence of a large clot of blood, or of a solid foreign body. I have said that spasmodic contractions might not act in a general way on the uterus. I will now add that this disease may, by affecting other parts of the body, cause the blood to flow towards the womb, and thereby occasion a violent hemorrhage, which would be promptly fatal, if not combated by vigorous means.

Perhaps it will be found, that what I have said with regard to the symptoms is a little diffuse, considering the work I have undertaken; and yet I am far from having exhausted the subject. But this consideration has not deterred me, believing that what I have said will not be without utility; because, when affections of so dangerous a character are the subjects of examination, the means of diagnosis cannot be too much multiplied. And even were I to have done nothing but copy the symptoms mentioned by other authors, I would still believe in the utility of this precaution, which will at least serve to refresh the memory of practitioners on this very important matter.

*Consequences of Hemorrhages.*—Hemorrhages, when not arrested, lead to anæmia, a condition which led M. Leyroux† to say, 1st. "That even moderate anæmia, succeeding to hemorrhage, leaves the patient on the verge of instantaneous and unexpected death, and in consequence enlists all the solicitude of the medical attendant. 2d. That the most trifling obstetrical operation may add to this debility a fatal perturbation,—a circumstance which must be kept in mind in determining upon the remedial measures to be employed."

The same Professor, replying to a refutation by M. Toux,‡ who finds that suggestion, if followed implicitly, would lead to consequences opposed to

* Case of Mrs. C. † Gazette des Hôpitaux, 22de Decembre, 1855.

‡ Gazette des Hôpitaux, 22 Janvier, 1856.

the true principles of the art of midwifery, and to the rules laid down in the immortal work of Baudelocque, in which it is said that inactivity in grave cases is a bad example to give to certain physicians, already too much inclined to this practice—M. Leyroux, I repeat, in his second article, expresses the wish that what he had said should not be understood as a rule to be followed, except in rare cases, of which he has collected and published a certain number in view of their practical utility.

I agree in many points with the distinguished physician of the Hôtel Dieu; but I cannot but object to several of his principles on this subject. I will content myself by giving several cases to point out the differences of our respective views. They will establish the rules which guide me in my practice.

*Case* 1. I was called, in 1845, to deliver a negro woman, slave to Mrs. L——, living in Havana. I found with this woman two young physicians, who, notwithstanding all the means employed by them, even to the use of the forceps, could not cause the labor to advance. The head was already in the lower basin. The abdomen being very large and lobulated, made me think that the pregnancy might be double, and that the two fœtuses might, by being engaged together, have occasioned a kind of wedging.

I examined the condition of things as carefully as the state of the genital organs, slightly tumefied, would permit, and I could only discover that the head was in a good position. In passing my hand over the abdomen, in the absence of pain, I felt, in the hypogastric region, a firm and circumscribed body, occupying the left iliac fossa, and resting on the pubis. This tumefaction, which felt like the head of a fœtus, was very distinct from that felt in the vagina. This reaffirmed me in my first opinion, that the pregnancy was double. I caused this body to be sustained during several expulsory efforts, but labor did not advance any faster than before.

I then decided on reapplying the forceps. After great traction-efforts the head was born. The tumor appeared to be dragged along with the head, for it was now hardly felt. Two fingers being introduced into the vagina, I was enabled to feel the head of the other fœtus pressed against the neck of the first. Labor having been already too long to justify the belief that the fœtus, whose head was already out, was alive, and the woman, also, saying that for several hours she had not felt the least motion, I thought it my duty to concentrate all my efforts on the head already born. My traction caused the second head to present itself. I discovered that my efforts would no longer be useful, and decided on applying the forceps to the second head. The application of the instrument, which I had thought would be very difficult, was, on the contrary, tolerably easy. The neck of the first fœtus, very narrow, was situated in the lower part of the vulva. I therefore drew out the head of the second fœtus. From this moment I

was enabled to ascertain that the necks of the two fœtuses were united at their bases, which left no room to doubt that I had to deal with a monstrosity, and, consequently, that the fœtuses must be drawn out together. After uniting the two heads, and covering them with a piece of fine linen, I applied moderate and methodic traction-efforts, by which the birth of the bodies of the fœtuses was obtained. In a few minutes more the afterbirth was expelled, and this being succeeded by the discharge of a quantity of blood, I concluded that the uterus was in a state of inertia. After so long a labor, after such immense efforts on the part of the uterus to expel these fœtuses, atony of this organ could scarcely fail to result. I made frictions on the hypogastric region and titillations on the neck; I compressed the abdominal aorta: nothing would arrest the hemorrhage. It was then that for the first time I thought of resorting to iodine injections, with a view to arrest puerperal hemorrhage; a treatment with which I had succeeded so well in combating those arising from other causes than gestation. I sent to a druggist residing near by for some tincture of iodine and a glass syringe with a long syphon terminating with an olive-shaped bulb pierced with several holes. I then injected into the cavity of the uterus half an ounce of the tincture of iodine, mixed with an ounce of water. The liquid was immediately rejected by the uterine contractions, and the uterus from that moment took on the condition natural after difficult labors. The patient was placed on tonics, and half a cup of broth every three hours. She fell asleep, and when she awoke said she was very well, but felt fatigued and weak. The lochial discharge was small, but of proper quality. From this moment nothing extraordinary occurred, and, with the exception of the weakness, which lasted some time, the consequences were as natural as those which usually result from artificial labor.

The fœtuses were united in front from the lower part of the neck to nearly the point of insertion of the umbilical cord. With this exception, each fœtus was perfectly formed: the genitals, the extremities, and the heads were natural. There was no deformity in the vertebral columns. The necks appeared to be united just above their base. Immediately underneath, the clavicles of each fœtus could be distinctly felt, but from this point to the lower part of the chest there was a complete adhesion of the true ribs. This adhesion, which appeared to be in the cartilaginous parts, presented a groove formed by the approximation of the convexities of the ribs, which extended to the lower part of the chest, and was exactly alike on both sides, and acted in such a way as to separate the two fœtuses as if to disjoin them. No motion could be obtained, which would not have been the case had the attachment been in the soft parts.

The placenta was visibly double, but united by means of a firm tissue, which was discoverable on the external surface by a prominent raphé. The placenta was oblong, but a little turned in at the centre of the two sides, towards the point of junction of which I have spoken. The points of insertion of the cords were at a short distance from each other.

This monster, which, according to Geoffroy de St. Hilaire, belongs to the Autositary class, was too interesting not to be made known. But, believing that the offer made by a professor of the medical school of this city to make its dissection would lead to more useful results than I could myself obtain, I gave it up to him, but, unhappily, all my efforts to obtain the result of his examination, or the fœtus, have been useless.

I trust that I shall be excused for the length to which these remarks have been carried, as well as for some of the details offered. These, I know, are foreign to the end proposed in these pages, but I could not resist the desire to make known this interesting case.

*Case* 2. Mrs. M——, 30 years of age, of a lymphatico-nervous temperament, had suffered during the last ten years (since the time of her only delivery), from a disease of the liver, when my professional advice was requested. The condition of this lady made me fear for her life; but by assiduous attention a degree of amendment, which I did not dare to hope for, was obtained. She so far recovered her health as soon to become pregnant. This new condition gave me great fears, more particularly as her father, Dr. A——, informed me that at her first labor he had considerable difficulties to overcome on account of the smallness of the transverse diameter of the lower basin, caused by the near approximation of the tuberosities of the ischia. But happily the smallness of the fœtus overcame this obstacle; very slowly, it is true, but without accident. A few months after, Mrs. M—— again became pregnant, but continued to be feeble. I hoped that this weakness, by being communicated to the fœtus, might prevent its full growth, and that by that means the labor would, like the last, terminate happily. The term of pregnancy passed over without any occurrence worthy of remark, but it was not thus at its close. I sent for a medical friend to assist me. After having ascertained the existence of the pelvic deformity of which I had been told, we found that the head was in the first position, and was very large and firm. We agreed that since the lady was very anæmic, and the strength she possessed would be insufficient to terminate the labor, and since, besides, it was necessary to prevent the loss of blood, we should apply the forceps as soon as the occasion would permit. This occasion did not present itself until that period of labor when a vigorous and well-constituted woman would have expelled the fœtus with one pain. In the meanwhile the patient grew weaker as well as cold at the extremities. These symptoms, combined with the occurrence of dyspnœa, made me believe in the existence of a hemorrhage. I sent immediately for some tincture of iodine and a syringe; the forceps were applied and a dead fœtus was extracted, followed by a considerable quantity of blood. The patient took two spoonfuls of brandy, and without losing time I introduced my hand in search of the placenta, which adhered but slightly. This manipulation was not followed by the contraction of the uterus. The iodine arrived, the injection was given at once, and the hemorrhage and inertia ceased.

I continued to combat the anæmic condition with tonics and broth; I also prescribed an etherial potion. I would be obliged to repeat what was mentioned in relation to the preceding case, if I wished to report what followed the labor of Mrs. M——, and I shall content myself with referring my reader to it, adding, however, that for twelve days after, I was obliged to draw off her urine three times a day, the bladder being too feeble to expel it.

*Case* 3. Mrs. C—— had given birth to a large child about eleven o'clock of the morning of the day on which I was sent for. I found in attendance two distinguished physicians of Havana. They having requested that a third physician should be sent for, I was, by chance, selected, and in a short time I reached the patient. I was told by the attendants that a considerable hemorrhage had followed the expulsion of the fœtus, and that they had in vain tried to remove the placenta, and cause a cessation of the inertia of the womb. Many means had been resorted to, but all ineffectually. One of the physicians continued to compress the abdominal aorta, notwithstanding which the blood continued to flow. Mrs. C——, of a lymphatico-nervous temperament, was in a most decided state of anæmia; she had frequent convulsions, dyspnœa, syncopies, cold sweats over the body. Her breathing was often accompanied with rattling. The pulse was very feeble and frequent, and disappeared at every fainting fit. I proposed injections of iodine, and, as the time required to obtain the medicine appeared to us very long, it was thought necessary to sustain her strength by giving her brandy or rum. I therefore made her take two spoonfuls, and, as the decision of the consultation was, that the placenta ought to be removed, I introduced my right hand guided by the cord held in the left. I encountered that part of it which had already been detached by one of the attendants. I continued to detach it, and six minutes had not elapsed before the delivery took place. But all the means employed to cause the womb to contract still proved useless; the uterine hemorrhage continued. The compression of the aorta was persevered in; alcohol, as well as linen steeped in water and vinegar, were applied to the hypogastric region. The tincture of iodine and the syringe having at last arrived, and being certain that no change for the better had taken place, the injection was resorted to in the same way as in the preceding cases, and the result was equally successful, causing the cessation of the hemorrhage and of the uterine inertia.

After having caused the patient to take two more spoonfuls of rum, and placed her in bed, I caused her to be wrapped up in hot flannels. Bottles of hot water were put almost in contact with her body. She was made to breathe ether or rum, and during the fainting fits she was directed to inhale ammonia. Her pulse was of incalculable frequency, very feeble, and at times ceased to beat. Notwithstanding symptoms so grave, I was not discouraged. The body of the patient was sprinkled over with starch and quinia. Half an hour after taking a spoonful of rum, she was given an equal quantity of syrup of ether; which was succeeded in half an hour by three spoonfuls of broth, and as much wine and water as she desired. One of the physicians having remained near the patient, it was observed that, after three hours of incessant care, she slept for ten minutes; her breathing during this time continued to be the same as before, hence the patient appeared to be rather in agony than asleep. She awoke with a start, as if suffocating; but the symptoms which followed this sleep did not appear to me to be so grave; the pulse became a little stronger, the fainting fits were not so frequent, and the patient could succeed in taking longer, and consequently less frequent inspirations, as she was often recommended to do. The remedies were continued as before, except that they were suspended during frequent little naps.

When I was satisfied that the patient was somewhat better, I asked for the afterbirth, which had been kept for me. I observed that the placenta was enormous, with a rent of about four inches long and a quarter of an inch deep, situated at about half an inch from the point of insertion of the cord. The latter, which was not less than half an inch in diameter, was hardly nine inches long. If we consider that the part remaining with the child ought to be about three inches, there would have still required twelve inches to make it of normal length.

After this examination, I asked myself to what the hemorrhage and inertia of the uterus, in Mrs. C——, could be attributed. I found a partial separation of the placenta, an extensive laceration of it, a very short cord, and an inertia of the uterus. As the hemorrhage followed the fœtus immediately, it could not be attributed to the partial separation of the placenta. The laceration on the contrary, appeared to have furnished the lost blood, but was not itself the consequence of the traction made during the extraction of the placenta; because, as I have said before, the hemorrhage occurred during the expulsion of the fœtus. I believe, therefore, that the laceration was the effect of the tension on the cord during labor: the insufficient length of it authorizes me to so believe. As to the inertia, it was, perhaps, the effect of the large size of the placenta; but the great loss of blood caused an asthenic condition, to which I attribute the continuance of this condition of the uterus.

Let us revert to our interesting patient, whom we left a little better. On my return, I found her still breathing too frequently; the fainting fits returned occasionally, but the sweats were less copious and less cold. The pulse was a little stronger, but still very frequent (150 a minute). The naps were a little longer, and less disturbed. The same medicines were continued. This state of uncertainty persisted during 72 hours; but after this the amendment of all the symptoms raised our hopes. The patient asked to see her child, whom she had not as yet inquired after. The stimulants were discontinued, but half a cup of broth was given every three hours, as also a decoction of dog-grass and of the capillary plant, as well as a little wine and water when she desired it. The milk fever was slight. The patient, continuing to improve every day, was allowed the diet suitable to women soon after delivery. The lactate of iron was prescribed and persevered in for two months. Three months after, the patient was still anæmic, and could take but little exercise, yet she gradually improved, and is now in her usual state of health.

In this, as in the preceding cases, the lochia were such as might be expected from the anæmic state of the patient. This, I believe, is a proof of the innocuity of the remedy which, besides, is so certain in its effects.

Are the remedial effects of iodine due to astringent or to exciting properties? If the action of astringents is due to a sort of puckering of the parts with which they are placed in contact; and if those of excitants, instead of producing the effects mentioned, stimulate the organic tissues, and render them more prompt in the exercise of their functions, there will naturally result from the latter an acceleration of the vital phenomena which, in the cases mentioned, were very much enfeebled by the asthenic state of the patients. Now we must admit that hemorrhages are arrested by virtue of an action substituted for the natural one, itself the product of excitation, which assumes the place of the exhausted vital forces, thereby provoking the contractions of the uterine tissues, and closing the vessels in such a manner as to prevent the escape of a large quantity of blood, and at the same time recalling and re-establishing the circulation to a degree commensurate with the strength of the patient.

I ought, perhaps, to have given a history of the hemostatic remedy here recommended, while describing the facts which have successively fallen under my observation. But I preferred to relate the most important cases, and to close with a few words on those which I consider so simple as hardly to merit being mentioned. For example:

A patient applies, laboring under chronic uterine hemorrhage, no matter how caused, whether by an organic affection or a functional derangement.

I place her under the following treatment: The woman is put upon an inclined plane, prepared across the bed by means of the back of a chair turned upside down and covered with pillows. The feet are placed on chairs, and the patient is seated on the edge of the bed, or rather on a pillow, which terminates the inclined plane. After having covered her with a sheet from the breast down, I direct her to raise up her clothes, so as to remain covered by the sheet alone. I then grease the trivalve speculum (a modification of Charrière's), and introduce it in the ordinary manner. Then I surround the instrument at its base with the edge of the sheet, which is crossed underneath, and tucked under the seat of the patient. I give the handles of the instrument to the patient herself to hold. By means of a candle I examine the state of the os uteri. The latter, which is situated in the middle of the instrument, is cleaned of the mucus which generally adheres to it. It then presents its orifice ready to admit the end of the syphon or the syringe. An instrument of this kind large enough to contain about an ounce and a half of fluid is selected for this operation. I have affixed to it a silver probe about five inches long; the end of the probe is a little enlarged, like that which is used to cauterize the Eustachian tube. The syringe is filled with a mixture of tincture of iodine and water, in the proportion of one-third of the former to two-thirds of the latter. The end of the syphon is pushed up, as far as possible, into the cavity of the uterus, and the injection administered with some degree of force. The liquid comes out of the cavity of the uterus as fast as introduced, and is soon all expelled. The uterus is ascertained to have contracted from the circumstance that mucus is very often attached to the end of the instrument in the form of a rather large ball. The woman experiences occasionally a sensation of heat, and sometimes a slight pain at the hypogastric region. The injection is repeated, if necessary, at the end of three or four days. The next day the hemorrhage is found to have lessened; it diminishes the following day, and generally disappears on the third.

I have resorted to this method of treatment more than a hundred times, and found but one case to resist it. This patient was an old lady, who had had uterine hemorrhage for many years. The innocuity of this method is too well exemplified by the preceding cases to render other facts necessary.

Having now proved the great usefulness of this method, to which I have no doubt the three individuals, whose cases are mentioned above, owe their lives, does it not become obligatory upon every accoucheur to have in his instrument case some tincture of iodine and a syringe, with a view of resorting to injections in cases of puerperal hemorrhage?

